# Inhibition of histone-deacetylase activity rescues inflammatory cystic fibrosis lung disease by modulating innate and adaptive immune responses

**DOI:** 10.1186/s12931-017-0705-8

**Published:** 2018-01-04

**Authors:** Manish Bodas, Steven Mazur, Taehong Min, Neeraj Vij

**Affiliations:** 1 College of Medicine, Central Michigan University, 2630 Denison Drive, Room# 120 (Office) & 126-127 (Lab), Mt Pleasant, MI USA; 2 Department of Pediatrics and Pulmonary Medicine, The Johns Hopkins University School of Medicine, Baltimore, MD USA; 3 National Institute of Allergy and Infectious Diseases, National Institutes of Health, Integrated Research Facility at Fort Detrick, Fort Detrick, Frederick, MD USA; 4 Genentech, 1 DNA Way, San Francisco, CA USA; 5 VIJ Biotech LLC, Baltimore, Maryland USA

**Keywords:** SAHA (suberoylanilide hydroxamic acid), Cystic fibrosis, CFTR, HDAC, Lung

## Abstract

**Background:**

Chronic lung disease resulting from dysfunctional cystic fibrosis transmembrane conductance regulator (CFTR) and NFκB-mediated neutrophilic-inflammation forms the basis of CF-related mortality. Here we aimed to evaluate if HDAC inhibition controls *Pseudomonas-aeruginosa-*lipopolysaccharide (*Pa*-LPS) induced airway inflammation and CF-lung disease.

**Methods:**

For in vitro experiments, HEK293-cells were transfected with IL-8 or NFκB-firefly luciferase, and SV40-renilla- luciferase reporter constructs or ΔF508-CFTR-pCEP, followed by treatment with suberoylanilide hydroxamic acid (SAHA), Trichostatin-A (TSA) and/or TNFα. For murine studies, *Cftr*^*+/+*^ or *Cftr*^*−/−*^ mice (*n* = 3) were injected/instilled with *Pa*-LPS and/or treated with SAHA or vehicle control. The progression of lung disease was monitored by quantifying changes in inflammatory markers (NFκB), cytokines (IL-6/IL-10), neutrophil activity (MPO, myeloperoxidase and/or NIMP-R14) and T-reg numbers.

**Results:**

SAHA treatment significantly (*p* < 0.05) suppresses TNFα-induced NFκB and IL-8 reporter activities in HEK293-cells. Moreover, SAHA, Tubacin (selective HDAC6-inhibitor) or HDAC6-shRNAs controls CSE-induced ER-stress activities (*p* < 0.05). In addition, SAHA restores trafficking of misfolded-ΔF508-CFTR, by inducing protein levels of both B and C forms of CFTR. Murine studies using *Cftr*^*+/+*^ or *Cftr*^*−/−*^ mice verified that SAHA controls *Pa*-LPS induced IL-6 levels, and neutrophil (MPO levels and/or NIMP-R14), NFκB-(inflammation) and Nrf2 (oxidative-stress marker) activities, while promoting FoxP3^+^ T-reg activity.

**Conclusion:**

In summary, SAHA-mediated HDAC inhibition modulates innate and adaptive immune responses involved in pathogenesis and progression of inflammatory CF-lung disease.

**Electronic supplementary material:**

The online version of this article (10.1186/s12931-017-0705-8) contains supplementary material, which is available to authorized users.

## Background

The loss of the membrane-localized cystic fibrosis transmembrane conductance regulator (CFTR) protein due to a loss-of-function mutation in the *Cftr* gene, leads to cystic fibrosis disease that severely affects the respiratory, gastrointestinal and reproductive organs [1–3]. The CF-related airway disease is a major contributor of the morbidity and mortality in CF patients. Although average survival age of CF subjects has substantially risen due to better clinical management of disease and availability of potent therapeutics that primarily target the symptoms of the disease, correction of the underlying genetic defect is needed to achieve the potential cure [4]. Clinically, CF-lung disease initiates as sustained airway inflammation and other complex abnormalities such as persistent infections, chronic inflammatory-oxidative stress and mucus hyper-secretion all contribute to the chronic airway obstruction [1, 2, 5]. The chronic exacerbation of CF involves repeated or stable infection with *Pseudomonas aeruginosa (Pa)* that results in chronic airway inflammation involving IL-8 mediated neutrophil chemotaxis resulting in irreversible pulmonary damage and respiratory failure [1, 2, 4, 5].

The most common genetic defect in CF is the deletion of phenylalanine-508 (ΔF508) in CFTR that results in misfolding of mutant CFTR protein and failure of CFTR ion channel to reach the plasma membrane; that results in ion channel dysfunction [1, 6]. This misfolded non-functional CFTR protein is either degraded by the ubiquitin proteasome system (UPS) [7] or aggregated as aggresome-bodies [8, 9], as autophagy is impaired [8, 10, 11]. The recent studies have shown that defective CFTR itself leads to ROS-mediated autophagy-impairment that contributes to the CFTR dysfunction and chronic inflammatory-oxidative stress observed in CF airways [8, 9]. Thus, the absence of CFTR on the cell surface results in chronic inflammation geared primarily by NFκB mediated pro-inflammatory signaling and IL-8-dependent neutrophil chemotaxis [1, 5]. The dominant pathogenic role of neutrophils in CF-lung disease is specifically attributed to the unopposed deleterious effects of neutrophil elastase on the structural lung epithelial cells that significantly contributes to lung damage and decreased pulmonary function in CF subjects [5, 12]. Apart from neutrophils, the other innate and adaptive immune cells also contribute to the pathophysiology of CF-lung disease by elevating levels of several pro-inflammatory cytokines and chemokines such as IL-1β, IL-6, IL-8, TNFα, IL-33 and IL-17 [13]. In addition, intriguing recent findings describe that an imbalance of Th17/T-reg cells contributes to the chronic inflammatory state seen in CF airways [14, 15], where pharmacological augmentation of T-reg numbers and/or their function can be used to control pathogenesis and/or progression of chronic lung disease.

It is important to note that, in addition to its classical channel function, lipid-raft or membrane-CFTR controls NFκB mediated inflammatory signaling that impacts both innate and adaptive immune responses in the CF airways [1, 16]. This suggests that future therapeutics targeting CF-lung disease should focus on rescuing misfolded ΔF508-CFTR to the plasma membrane while concurrently suppressing NFκB mediated hyper-inflammatory responses [1]. To this end, the pharmacological modulation of histone deacetylases (HDACs) seems promising, as HDACs regulate the expression of genes involved in inflammation and apoptosis through deacetylation of histone or non-histone proteins, in addition to rescuing the underlying CF defect. In support of this concept, the therapeutic potential of HDAC inhibitors (or proteostasis-modulators), in rescuing ΔF508-CFTR to the plasma membrane is clearly demonstrated [3, 17]. In addition, the therapeutic and clinical efficacy of HDAC-inhibition (HDACi) in numerous other disease states such as inflammatory bowel disease (IBD), cancer, AIDS, graft-versus-host disease and rheumatoid arthritis warrants its further preclinical evaluation in controlling pathogenesis of CF-lung disease.

Thus, we aimed to evaluate the therapeutic utility of HDACi in controlling *Pa*-LPS induced neutrophilic lung inflammation in CF-preclinical murine models (*Cftr*^*+/+*^ and *Cftr*^*−/−*^ mice). Our data suggests that inhibition of HDAC activity can control *Pa*-LPS induced lung disease by modulating NFκB-mediated inflammatory signaling, neutrophil chemotaxis and T-reg-activation by CFTR-dependent and/or independent mechanisms. The HDACi-governed mechanisms of innate and adaptive immune response modulation described in this study highlight the scope of a novel intervention strategy for controlling CF-lung disease or other chronic conditions.

## Methods

### In vitro experiments

The human embryonic kidney cells (HEK293) were cultured using standard cell culture conditions, as previously described [18]. Cells were transiently transfected with firefly luciferase– IL-8 or NFκB, and SV40-renilla luciferase- reporter plasmid constructs followed by overnight treatment with suberoylanilide hydroxamic acid (SAHA, 10 μM), Trichostatin-A (TSA, 10 μM) and/or TNFα (10 ng/ml). For ER-stress reporter assays, cells were transfected with a secretory gaussia reporter plasmid, pSM2 and/or HDAC6 shRNA for 36–48 h; with parallel overnight treatment of Tubacin (10 μM) and/or cigarette smoke extract (CSE). The CSE was prepared as recently described [8, 10] and the reporter activities were assayed by commercially available Dual-Luciferase® Reporter System (Promega). In a separate experiment, HEK293 cells were transfected with HDAC6 shRNAs, pCEP-ΔF508CFTR or control plasmids (for 24 h) and treated with SAHA or TSA for final 24 h followed by immunoblotting for HDAC6 and β-actin or metabolic S^35^-labeling, CFTR immunoprecipitation and radiography as previously described [18].

### Murine experiments

All animal experiments were performed as per the JHU Institutional Animal Care & Use Committee (IACUC) approved protocol and guidelines. To standardize the in vivo dose of SAHA, age and sex matched *Cftr*^*+/+*^ mice (C57BL6 WT mice) were intratracheally (i.t.) treated with *Pseudomonas aeruginosa* lipopolysaccharide *(Pa-*LPS) (20 μg/mouse, *n* = 3). After 12 h post *Pa*-LPS challenge, the mice were i.t. treated with SAHA (100 μg/mouse in 100 μl total volume of PBS) for one (SAHA-A), two (SAHA-B) or three (SAHA-C) days. In the following experiment, *Cftr*^*+/+*^ mice were i.t. treated with SAHA (50 μg/mouse in 100 μl total volume of PBS), for 12 h, post *Pa*-LPS challenge, for another 24 h. For the cigarette smoke (CS) exposure studies, age and sex matched *Cftr*^*+/+*^ mice (*n* = 3) were separated into four experimental groups: a) room-air, b) SAHA (50 μg/mouse, 3 total doses with 1 day interval before the termination of the experiment), c) sub-chronic CS (sc-CS), and d) sc-CS + SAHA. The room-air or side-stream CS exposures were performed for 8-weeks (sub-chronic exposure model) following our recently described protocol [8, 10, 19] and BALF cells were harvested at the termination of the experiment for quantitative flow cytometry analysis as described below. To evaluate the *Pa*-LPS induced CF-lung disease, we performed parallel experiments using the gut-corrected *Cftr*^*−/−*^ mice (*n* = 3, each group), which were procured from Case Western Reserve University Animal Resource Center, Cleveland, OH [16, 20]. These mice were injected intrperitoneally (i.p.) with SAHA (25 mg/kg bw) and/or *(Pa-*LPS) (15 mg/kg bw) for 36 h. Mice were sacrificed and the serum (to evaluate systemic responses) and lung tissues (to evaluate pulmonary responses) were harvested for further experimentation as described below. No significant changes in mice body weight or survival rate were observed during the time course of this experiment.

### Immunoblotting, fluorescence microscopy and pulse chase

We used our previously described immunoblotting method [10, 16, 20] to quantify changes in Nrf2, NFκB, CFTR and β-actin in total protein lysates isolated from cells or murine-lung tissue. All antibodies were purchased from Santa Cruz Biotechnology (scbt), except β-actin, which was from Sigma. Image J software was used to quantify the changes in protein expression relative to β-actin (loading control). For fluorescence microscopy, paraffin-embedded longitudinal lung tissue sections (5 μM) were prepared from all four experimental murine groups (Control, *Pa*-LPS, SAHA and *Pa*-LPS + SAHA). The lung sections were de-paraffinized and immunostained using our previously described protocol [10, 16]. The primary antibodies used for immunostaining were NFκB (rabbit polyclonal, scbt) and NIMP R14 (rat monoclonal, Abcam), followed by secondary staining with goat anti-rabbit IgG FITC (scbt) and goat anti-rat IgG (H + L) R-PE secondary antibodies. The Hoechst dye (Invitrogen) was used to stain the nuclei as described before [8, 10]. For pulse chase experiment, HEK293 cells were transiently transfected with pCEP-ΔF508CFTR plasmid and treated with SAHA (10 μM) or Trichostatin-A (10 μM) for 48 h. After 48 h of transfection, cells were pulsed with 250 μCi/well Trans-S^35^-cys/met (ICN Biomedicals Inc., Irvine CA) for 30 min. Next, cells were washed with PBS (1X) and 1 ml selective media (MEM) was added followed by chase for the indicated time points to evaluate the protein processing of radiolabeled CFTR. Briefly, the lysates were immuno-precipitated with CFTR 169 antibody as described above and run on SDS-PAGE and an autoradiography was performed as previously described [18].

### ELISA, MPO assay and flow cytometry

The inflammatory state of the mice exposed to *Pa*-LPS and/or SAHA was quantified by measuring BALF or serum cytokine levels (IL-6) and neutrophil (MPO, myeloperoxidase) activity by sandwich ELISAs (R&D and Hycult Biotechnology respectively), as recently described [10, 16]. The flow cytometry analysis of BALF samples from each murine experimental group was performed as previously described [10, 16, 21] and was used to quantify the percentage changes in number of CD4 + FoxP3+ T cells in BALF-cells. The BD FACS Caliber instrument and BD Cell Quest Pro software was used for acquisition and analysis of the data.

### Statistical analysis

The data is presented as mean ± SEM (or SD, as indicated) of each experimental group. A two-tailed unpaired Student’s t-test was performed to determine the significance between each data set and a *p*-value ≤0.05 was considered a significant change, where ‘*’ depicts *p* < 0.05, ‘**’ *p* < 0.01 and ‘***’ *p* < 0.001. For immunoblotting data, densitometry was used to quantify changes in each experimental group, using the Image J software (NIH).

## Results

### HDAC inhibition controls TNFα-induced IL-8 and NFκB promoter activities

**Fig. 1:**
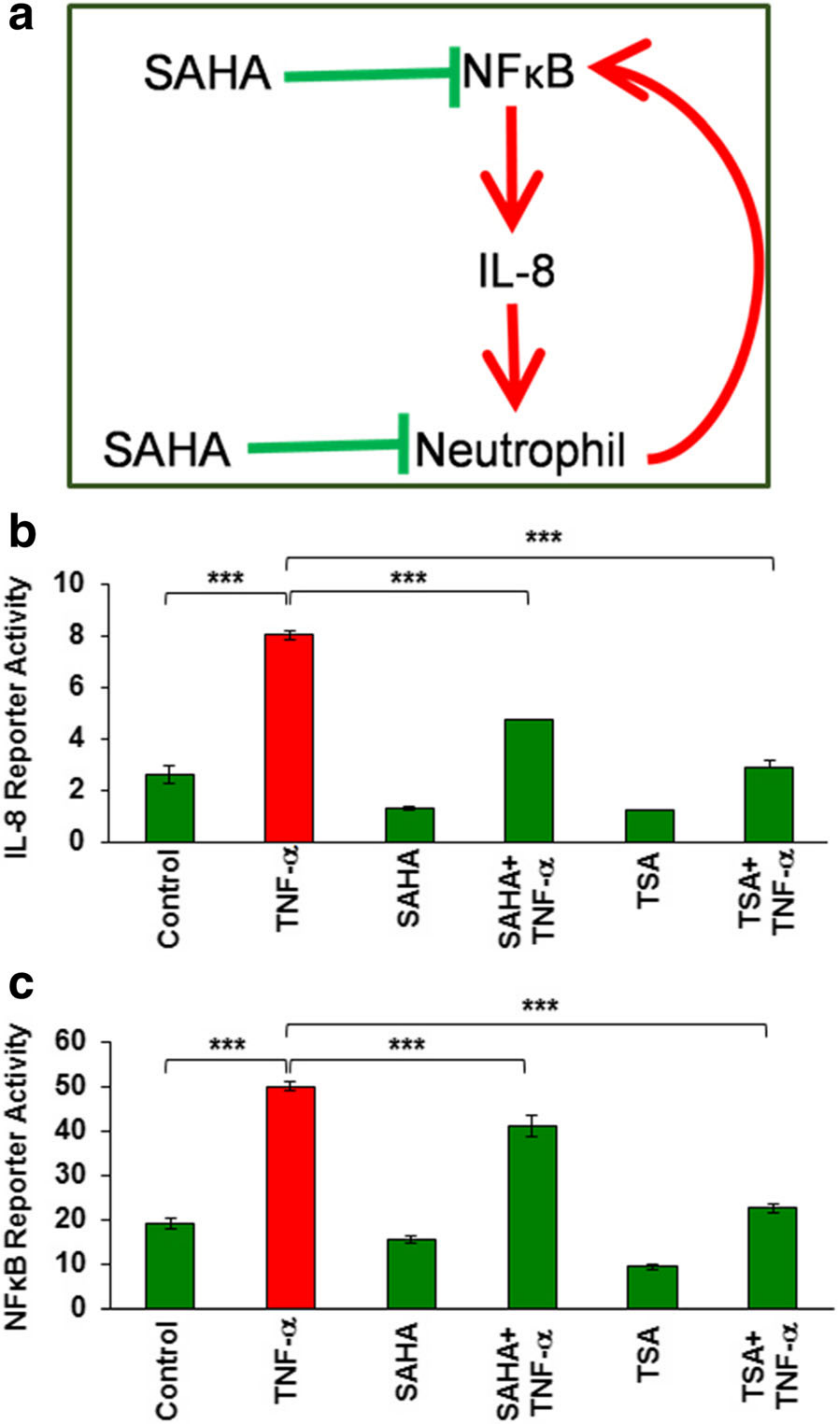
SAHA and Trichostatin-A (TSA) inhibits TNFα induced IL-8 and NFκB promoter activities. **a** Previous studies show that HDAC inhibition (HDACi) using SAHA has an anti-inflammatory action by controlling NFκB mediated neutrophil recruitment and activity. Thus, SAHA is anticipated to subvert the vicious inflammatory response governed by the NFκB-neutrophil-NFκB axis. **b, c**. Based on this prior notion, we first verified if HDACi could control TNFα (inflammatory cytokine)-induced IL-8 and NFκB reporter activities. HEK293 cells were transiently transfected with firefly IL-8 (**b**) or NFκB (**c**) reporter constructs with a renilla-luciferase internal control plasmid (*n* = 3). Cells were induced with TNFα (10 ng/ml) for 16 h to stimulate pro-inflammatory signaling. IL-8 and NFκB luciferase activities were normalized to the internal renilla luciferase control and data are shown as mean ± SD. We observed that treatment with SAHA (Class I/II HDAC inhibitor, 10 μM) and TSA (Class I/II HDAC inhibitor, 10 μM) significantly inhibits (*p* < 0.05) both basal and TNFα-induced IL-8 and NFκB activities, indicating that HDACi can ameliorate IL-8 and NFκB mediated inflammation and hence has the potential to control cystic fibrosis (CF) lung disease

CFTR dysfunction and chronic airway inflammation drives the pathogenesis of CF-lung disease [13, 22]. Previous studies in other chronic inflammatory conditions have reported that SAHA mediated HDAC-inhibition (HDACi) controls NFκB signaling and neutrophil recruitment and/or activity [23–25]. Thus SAHA is anticipated to help disrupt the vicious chronic cycle of inflammatory signaling regulated by the NFκB-neutrophil-NFκB axis in CF (Fig. 1a). Since TNFα is an important mediator of inflammatory signaling in chronic airway diseases, including CF [26], we first evaluated if HDACi using SAHA or Trichostatin-A (TSA) controls TNFα induced inflammation. Our results show that both SAHA and TSA significantly inhibits (*p* < 0.05) TNFα-induced NFκB and IL-8 reporter activities (Fig. 1b and c), suggesting that HDACi has the potential to control TNFα-induced inflammation and/or chronic CF-lung disease. Additionally, the retention of ΔF508-CFTR in the endoplasmic reticulum (ER) leads to chronic ER-stress [27, 28] that mediates NFκB-mediated chronic airway inflammation in the CF lungs [1]. Here, we demonstrate that HDACi using SAHA (or tubacin), or shRNA mediated HDAC6-knockdown, significantly controls cigarette smoke extract (CSE) exposure induced ER-stress activity (Additional file 1: Figure S1A and B), suggesting the potential of selective HDACi in reducing the misfolded ΔF508-CFTR induced ER-stress in the CF-airways.

### SAHA controls *Pa*-LPS induced lung inflammation and neutrophil activity

**Fig. 2:**
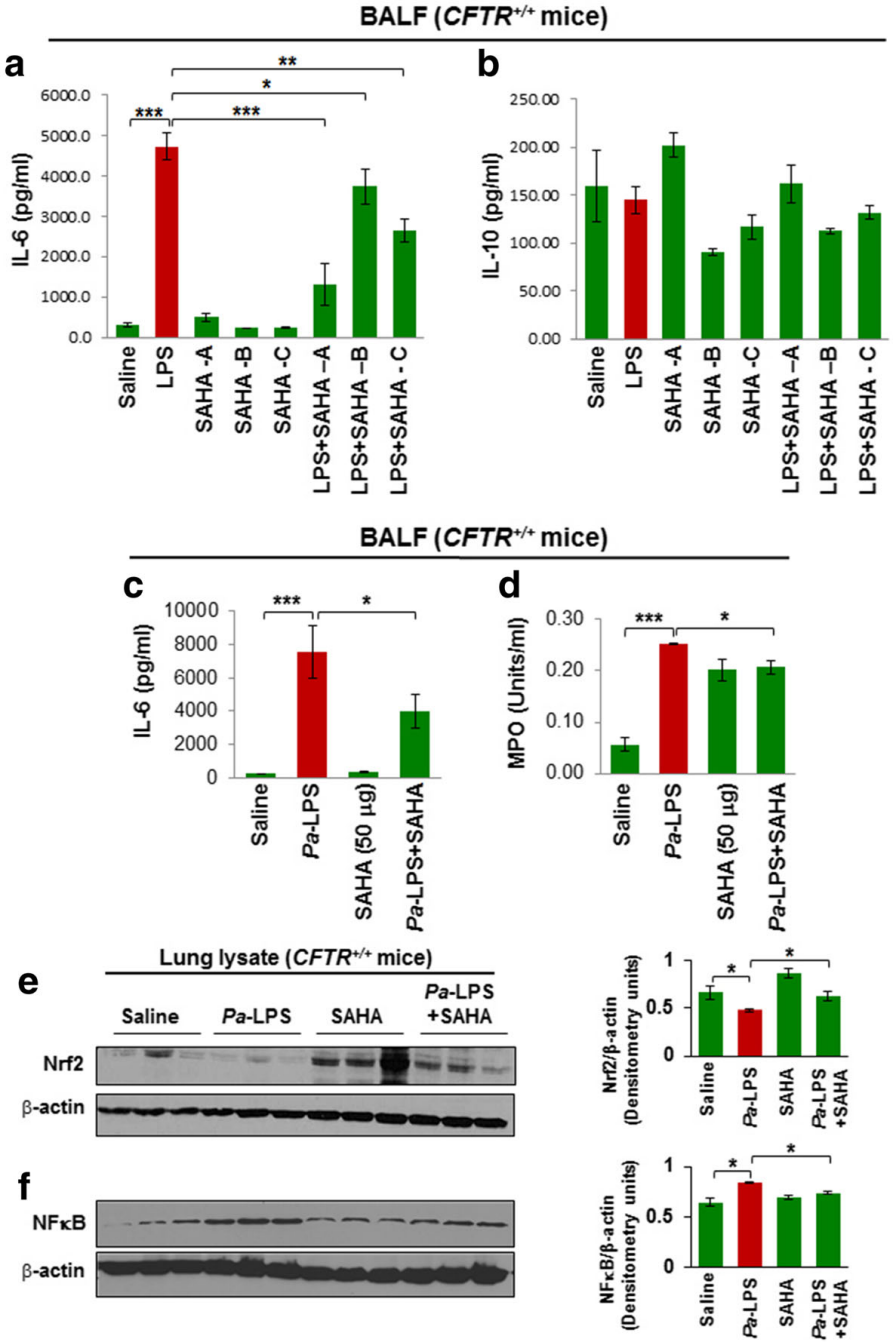
SAHA protects against *Pseudomonas aeruginosa-*LPS (*Pa*-LPS) - induced inflammatory lung disease. **a, b** The *Cftr*^*+/+*^ mice were intra-tracheally (i.t.) instilled with *Pa*-LPS (20 μg/mouse, n = 3), followed by SAHA treatment (100 μg/mouse in 100 μl total volume of PBS) for one (SAHA-A), two (SAHA-B) or three (SAHA-C) days, in order to standardize the correct dose and duration of SAHA treatment. The bronchoalveolar lavage fluid (BALF) was collected each day and analyzed for IL-6 and IL-10 cytokine levels by ELISA. Data shows that treatment with SAHA significantly (*p* < 0.05) controls *Pa*-LPS induced IL-6 levels at day 1, although the rescue was not very significant at day 2 or 3, implying that repeated SAHA administration might have some side effects. Moreover, even though no significant change was detected in IL-10 levels in BALF isolated from *Pa*-LPS treated mice compared to controls, we did observe a slight decrease, which was partially restored by SAHA treatment (Day 1), indicating that further standardization of SAHA dose is needed. **c, d** Based on the above preliminary data, we treated *Cftr*^*+/+*^ mice with i.t. SAHA (50 μg/mouse in 100 μl total volume of PBS, *n* = 3), for 12 h, post *Pa*-LPS (20 μg/mouse) challenge for another 24 h, to test the therapeutic efficacy of a lower SAHA dose. The BALF was harvested and analyzed for IL-6 (inflammation) levels and neutrophil (myeloperoxidase, MPO) activity. The data shows that even a lower dose (50 μg/mouse) of SAHA significantly controls *Pa*-LPS induced inflammation (IL-6) and neutrophil activity (MPO), suggesting it’s potential in controlling *Pa*-LPS induced pulmonary inflammation or lung disease. **e, f** Next, the lung tissues harvested from the above experiment (C) were used for immunoblotting. The data shows that SAHA treatment controls *Pa*-LPS induced inflammatory-oxidative stress by regulating Nrf2 and NFκB protein expression (*p* < 0.05)

The CF pathogen, *Pseudomonas aeruginosa* (*Pa)*, is known to contribute significantly to the chronic inflammation that drives the progression of CF-lung disease [1, 13, 22]. Treatment with LPS derived from *Pa* (*Pa*-LPS), is a widely used in vivo model for mimicking CF-related inflammation and lung disease [16, 29]. We first used *Cftr*^*+/+*^ mice treated with *Pa*-LPS as a model of CF-related lung inflammation. Our initial goal was to verify the suitable in vivo dose and duration of SAHA treatment for controlling *Pa*-LPS induced lung inflammation. Thus, we i.t. treated these mice with *Pa*-LPS (20 μg/mouse) followed by three daily doses of SAHA (100 μg/mouse). We found that SAHA treatment significantly (*p* < 0.05) controlled *Pa*-LPS induced IL-6 levels in mice treated with SAHA for one day (SAHA-A), while two (SAHA-B) or three (SAHA-C) days of treatment seemed to have detrimental effects, with little control of IL-6 mediated inflammatory state as compared to one day treatment (Fig. 2a). Next, we evaluated the changes in levels of IL-10, a regulatory T cell (CD4 + FoxP3+ T-regs)-associated immunosuppressive cytokine [30], as T-reg dysfunction is implicated in chronic CF-lung disease pathogenesis [14]. We found that although *Pa*-LPS could not significantly decrease IL-10 levels, the basal IL-10 level is partially elevated by SAHA-A, but not by SAHA-B/C (Fig. 2b), implying that SAHA induced IL-10 (or T-regs) could possibly check *Pa*-LPS mediated inflammation on further standardization of dose. Since we observed that two or 3 days of SAHA treatment (100 μg/mouse) was not as effective in controlling inflammation as one day treatment (Fig. 2a), we tested the efficacy of a lower dose of SAHA (50 μg/mouse) in controlling *Pa*-LPS induced airway inflammation. The data shows that *Pa*-LPS induced IL-6 and myeloperoxidase (MPO) levels are significantly reduced by SAHA (Fig. 2c–p<0.05) suggesting the potential of HDACi in controlling *Pa*-LPS induced chronic airway inflammation and neutrophil activity. Additionally, we also found that *Pa*-LPS induced NFκB activation (inflammation) and Nrf2 down-regulation (anti-oxidant response) are significantly corrected by SAHA, indicating towards the mechanism of anti-inflammatory action of SAHA in the murine lungs (Fig. 2e–p<0.05). Overall, data suggests the therapeutic potential of SAHA (or other specific HDAC inhibitors) in rescuing *Pa*-LPS induced CF-lung disease via partial induction of IL-10 (T-reg associated cytokine), while inhibiting neutrophil influx.

### SAHA restores *Pa*-LPS induced decrease in regulatory T cells

**Fig. 3:**
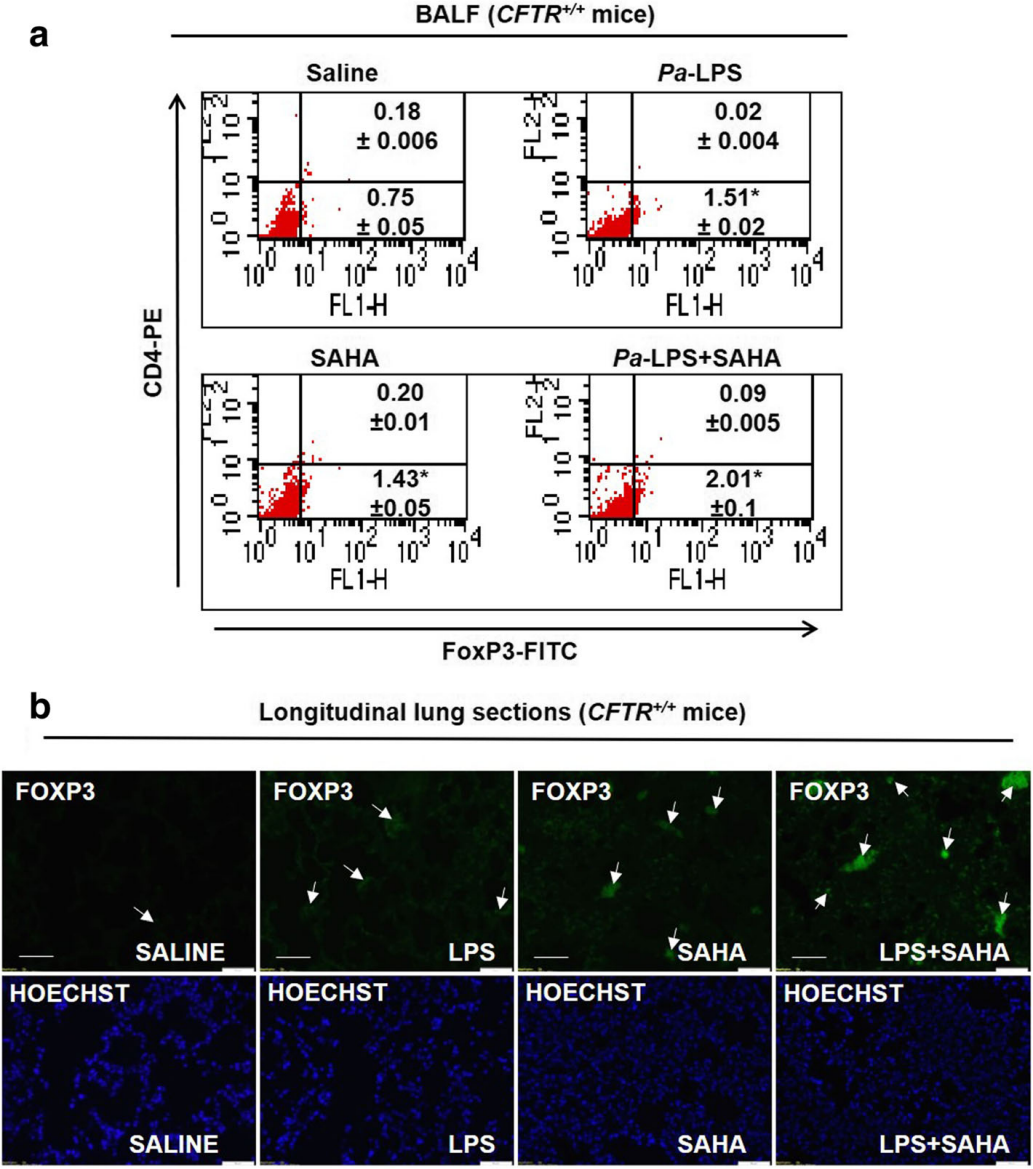
SAHA restores *Pa*-LPS mediated decrease in FoxP3+ T-reg cells. The *Cftr*^*+/+*^ mice were i.t. instilled with *Pa*-LPS (20 μg/mouse) for total of 36 h (*n* = 3). Mice were i.t. treated with SAHA (50 μg/mouse in 100 μl total volume of PBS), for 12 h post *Pa*-LPS challenge. **a** The BALF cells were harvested and analyzed for CD4+ (CD4-PE antibody) and FoxP3+ [(FoxP3 (Rabbit polyclonal primary Ab)-Anti-Rabbit-FITC (secondary Ab)] cells by flow cytometry using the BD FACS Caliber instrument. Data indicates that SAHA treatment significantly (*p* < 0.05) induces the levels of FoxP3+ T regs to counteract the *Pa*-LPS mediated lung inflammation. The data represents mean ± SD from three independent experiments. **b** Immunostaining of longitudinal lung sections from the same groups of *Cftr*^*+/+*^ mice show that SAHA induces FoxP3 expression (white arrows) that implicates elevated T-reg numbers in the lungs to protect against *Pa*-LPS induced inflammatory disease

To verify whether SAHA augments T-reg cell numbers, *Cftr*^*+/+*^ mice were treated with *Pa*-LPS and/or SAHA as indicated. Our data shows that both *Pa*-LPS and SAHA treatments induced a significant increase in the percentage of FoxP3+ T cells as compared to saline treated control. This was marginally induced further in the *Pa*-LPS + SAHA group indicating that SAHA controls *Pa*-LPS mediated airway inflammation by elevating T-reg cell numbers that suppresses the hyper-inflammatory cells in the pulmonary microenvironment (Fig. 3a, *p < 0.05). Similar to our observation, other studies have also seen that SAHA’s anti-inflammatory action involves induction of FoxP3+ T reg cells [31]. The immunostaining of mice lung tissue sections from the same experimental groups verifies that SAHA induces expression of FoxP3+ cells (white arrows), suggesting an increase in the number of T-regs as a mechanism to suppress *Pa*-LPS induced pulmonary inflammation (Fig. 3b).

### SAHA controls *Pa*-LPS induced CF-lung disease

**Fig. 4:**
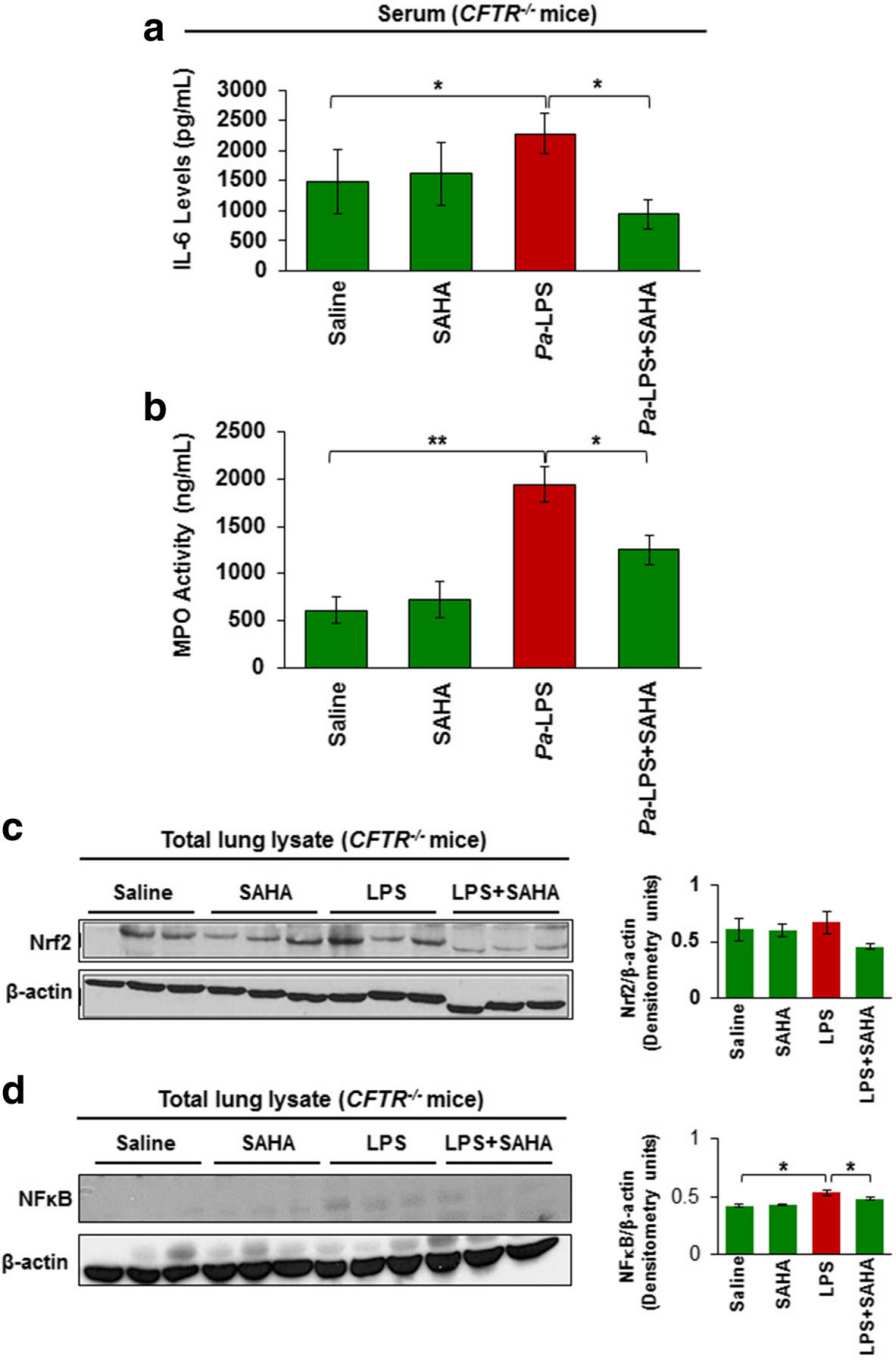
SAHA controls *Pa*-LPS induced inflammation and neutrophil activity in *Cftr*^*−/−*^ mice. The age and sex matched *Cftr*^*−/−*^ mice (n = 3, each group) were injected (i.p.) with SAHA (25 mg/kg bw) and/or *Pa-*LPS (15 mg/kg bw) for 36 h and serum was collected for further analysis. **a** IL-6 cytokine levels were quantified by sandwich ELISA. Data are shown as mean ± SEM of IL-6 levels (pg/ml). We observed that SAHA significantly inhibits (p < 0.05) *Pa*-LPS induced IL-6 cytokine levels. **b** To obtain a quantitative estimate of neutrophil recruitment and activity, a myeloperoxidase (MPO, neutrophil specific enzyme) ELISA assay was performed. We found that SAHA significantly inhibits (p < 0.05) *Pa*-LPS induced MPO (neutrophil activity) levels where data is shown as mean ± SD (ng/ml). **c, d** The age and sex matched *Cftr*^*−/−*^ mice (n = 3, each group) were injected (i.p.) with SAHA (25 mg/kg bw) and/or *Pa-*LPS (15 mg/kg bw) for 36 h, and lung tissues were collected for immunoblotting. Data shows that SAHA modulates *Pa*-LPS induced NFκB and Nrf2 protein levels, suggesting that SAHA mediated HDACi can be developed as an effective treatment for treating chronic CF-lung disease

**Fig. 5:**
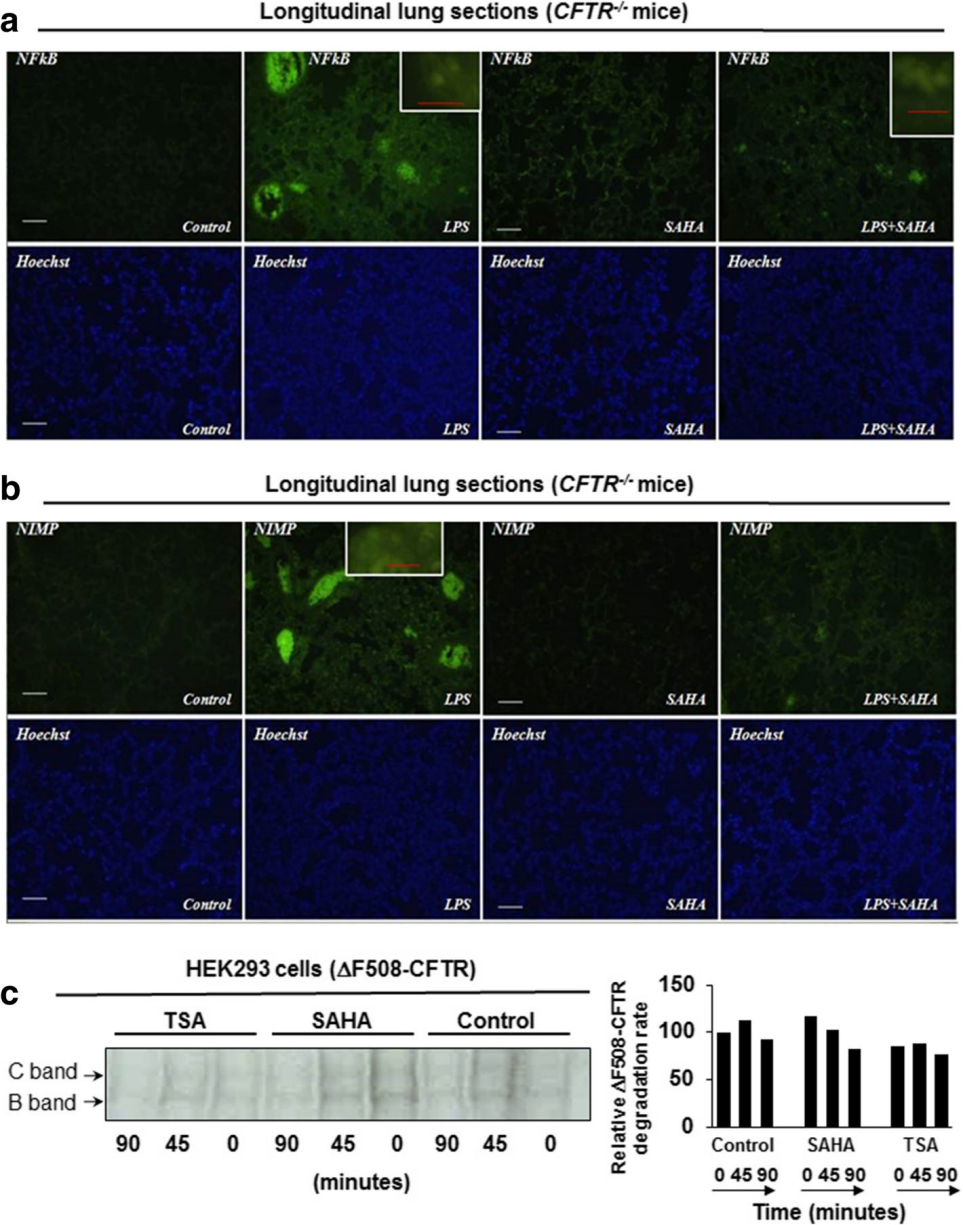
SAHA inhibits *Pa-*LPS induced NFκB activity and neutrophil chemotaxis. The age and sex matched *Cftr*^*−/−*^ mice (n = 3, each group) were injected (i.p.) with SAHA (25 mg/kg bw) and/or *Pa-*LPS (15 mg/kg bw) for 36 h, and lung tissues were collected for immunostaining. **a** SAHA treated *Cftr*^*−/−*^ mice show substantial reduction in *Pa*-LPS induced NFκB expression and nuclear localization in the murine lungs. **b** SAHA treatment also shows a substantial decline in the number of *Pa*-LPS driven neutrophils as depicted by NIMP R14 (a cell surface neutrophil marker) expression. Bottom panel shows the Hoechst (nuclear) staining and high-magnification images are shown in insets. Scale- 50 μM, insets-10 μM. **c** Next, HEK293 cells were transiently transfected with CEP-ΔF508-CFTR and treated with SAHA (10 μM) or Trichostatin A (10 μM) from 24 to 48 h. At 48 h post-transfection, cells were metabolically labelled with Trans-S^35^ for 30 min followed by 0, 45, and 90 min chases for evaluating the protein processing of radiolabelled CFTR by immunoprecipitation and autoradiography. The data shows that SAHA induces the expression of both the immature (B-form) and mature (C-form) forms of CFTR. Moreover, kinetic analysis suggests that SAHA slows the degradation rate of ∆F508-CFTR over untreated control and Trichostatin-A treatment. The data is representative of three independent experiments, *n* = 1

The gut-corrected *Cftr*^*−/−*^ mice (CF-mice) is a commonly accepted model for investigating the underlying mechanisms of CF-lung disease progression or evaluating the therapeutics for treating CF-lung disease [16, 32]. Thus, we used *Pa*-LPS treatment of CF-mice to assess the efficacy of SAHA in controlling *Pa*-induced inflammatory CF-lung disease. We found that SAHA significantly (*p* < 0.05) diminishes *Pa*-LPS induced systemic IL-6 and MPO levels (Fig. 4a and b), suggesting its potential as a therapeutic for controlling chronic inflammation and neutrophil-activation mediated obstructive CF-lung disease. Moreover, we also found that SAHA significantly inhibits *Pa*-LPS induced NFκB-activation (Figs. 4d and 5a, p<0.05) and neutrophil infiltration (NIMP-R14 immunostaining, Fig. 5b) in *Cftr*^*−/−*^ murine lungs, suggesting a CFTR independent mechanism. There was no statistically significant change in Nrf2 levels as determined by normalizing with β-actin using densitometry analysis, even though visual interpretation shows a decrease in Nrf2 levels in LPS+SAHA group (Fig. 4c). Apart from the classical genetic cause of membrane-CFTR deficiency, cigarette smoke (CS) exposure (acquired CFTR-dysfunction) also leads to diminished lipid-raft expression of CFTR, resulting in chronic inflammatory-oxidative stress in the airways [8]. Hence, we demonstrate here that HDACi using SAHA can also restore sub-chronic CS (sc-CS) mediated decrease in T-reg cell numbers (Additional file 1: Figure S1C), suggesting that SAHA treatment might be beneficial in controlling inflammation in disease states with acquired CFTR-dysfunction, such as chronic obstructive pulmonary disease (COPD). Thus, our data suggests that HDACi using SAHA or other more specific HDAC inhibitors such as Tubacin has the potential to rescue CF-related chronic inflammation and lung disease pathogenesis.

### SAHA induces ΔF508-CFTR trafficking by inhibiting its proteasomal degradation

To verify if SAHA has any role in modulating the proteostasis of CFTR-protein, we performed a pulse chase experiment as described previously [33], and found that SAHA induces the levels of both mature (C-band) and immature forms (B-band) of CFTR and delays the degradation rate of ΔF508-CFTR as compared to untreated control or TSA treatment (Fig. 5c). This implies that SAHA functions as a proteostasis-modulator and facilitates the proper folding of CFTR protein leading to the increased levels of the mature functional form of CFTR, i.e. called the C-form. The data corroborates the previous findings [3, 17, 34], which demonstrated the potency of HDAC inhibition in rescuing functional ΔF508-CFTR to the plasma membrane. We demonstrate here that HDACi by SAHA can not only correct ΔF508-CFTR proteostasis defect and/or function but also control pathogenesis of chronic obstructive lung disease by directly targeting IL-8-mediated neutrophil- chemotaxis and augmenting T-regs.

## Discussion

Cystic fibrosis lung disease is a hereditary condition caused due to the presence of ΔF508-CFTR, the misfolded form of the CFTR-protein, and the resulting diminished expression of functional WT-CFTR on the plasma membrane that leads to autophagy-impairment and elevated chronic inflammatory-oxidative stress responses [1, 6, 8, 9, 16]. These factors contribute significantly to the pathophysiological manifestations of CF-lung disease causing chronic and irreversible airway inflammation geared by invasion of several immune-inflammatory cells into the CF-airways [13]. Moreover, the pathogenic CF-*Pseudomonas aeruginosa* exacerbation further complicates the disease by recruitment and activation of additional inflammatory cells, especially neutrophils, into the airways [5, 12]. Thus, given the prominent role of IL-8-dependent neutrophil recruitment and activation in inflicting structural damage to the lung parenchyma, controlling neutrophilia is an important strategy for controlling CF-lung disease progression [12, 35]. Additionally, other immune cells such as T-helper type 2 (Th2) cells and macrophages also play a vital role in governing the chronic inflammatory response-mediated lung disease progression in the CF-airways [13]. Moreover, an imbalance between Th17 (inflammatory cells) and regulatory-T cells (T-regs, immunosuppressive cells) has been implicated in CF pathogenesis [14], which also provides a potential therapeutic strategy for suppressing CF lung inflammation. In the present study, we demonstrate the utility of SAHA (suberoylanilide hydroxamic acid, a HDAC inhibitor), in modulating innate and adaptive immune responses by controlling NFκB-IL-8-mediated neutrophil-chemotaxis, and T-reg activation, which may control the progression of chronic CF-lung disease by balancing the inflammatory response.

Numerous studies have documented the therapeutic benefits of SAHA in controlling tumorigenesis and inflammatory or auto-immune disease states such as RSV infections, septic shock, ventilator-induced lung injury, and inflammatory bowel disease (IBD) [23, 24]. Mechanistically, SAHA’s inflammation quenching function is due to its ability to control NFκB-mediated pro-inflammatory cytokine (TNFα, IL-1β, IL-8) response and myeloperoxidase (MPO, neutrophil specific enzyme) activity (Fig. 1a) [23, 24]. Our present data validates these findings as treatment with SAHA suppresses TNFα- induced IL-8 and NFκB activities (Fig. 1b, c). We used *Pa*-LPS instillation into the murine lungs to trigger inflammatory-oxidative stress as observed in the CF-airways and demonstrated here the pharmacological potential of SAHA in controlling the *Pa*-LPS induced inflammatory cytokine, IL-6 and MPO (neutrophil activation marker, Fig. 2a-d) levels, suggesting its anti-inflammatory potential. Moreover, SAHA is also reported to act as an antioxidant, as it suppresses LPS-mediated reactive oxygen species (ROS) activity and elevates glutathione levels, which is a potent anti-oxidant [36]. Our data supports this hypothesis as we observed that SAHA modulates *Pa*-LPS mediated changes in the anti-oxidant Nrf2 (in *Cftr*^*+/+*^ mice) and NFκB (inflammation) (Fig. 2e, f and Fig. 4c, d), which indicates towards its potential utility in controlling inflammatory-oxidative stress in the CF-airways that requires further experimental validation. In fact, some previous studies have clearly shown that the CF-epithelial cells have diminished Nrf2 protein levels (especially in the nucleus), reduced transcriptional activity, as well as insufficient expression of Nrf2 target genes, as compared to non-CF cells [22, 37–39]. This supports our notion that SAHA-mediated increase in Nrf2 levels might be beneficial in controlling inflammatory-oxidative stress in CF. Moreover, infection-mediated airway inflammation in CF is also known to trigger the unfolded protein response (UPR), a form of endoplasmic reticulum stress (ER stress) that is implicated in the pathology of CF-lung disease [40]. In the present study, we used cigarette smoke extract (CSE) - induced in vitro model of ER-stress to demonstrate that HDAC inhibition by SAHA, Tubacin (a specific HDAC6 inhibitor) or HDAC6-knockdown, controls CSE-induced ER-stress (Additional file 1: Figure S1A and B) and resulting inflammatory response, verifying its added pharmacological potential in controlling the CF-lung disease.

Since chronic inflammation involves aberrant activation of immune cells that governs CF-disease pathogenesis [13], it is not surprising that the immunosuppressive regulatory T cells (T-regs) that maintain immune homeostasis, are suggested to play a crucial role in CF lung pathophysiology [41]. A recent study shows that in CF patients with chronic *Pa* infection, there is an age-dependent, qualitative and quantitative impairment of T-regs [14]. Moreover, in children with CF, a decreased expression of CD4^+^CD25^+^FOXP3^+^ T cells (T-regs) correlates with lung function decline (FEV1) [42] suggesting that deficiency of T-reg numbers and/or function contributes to the chronic inflammatory state of CF-airways. Here, we verify this hypothesis by showing that SAHA treatment augments the number of FOXP3^+^ T cells (Fig. 3a, Additional file 1: Figure S1C). Our data verifies that SAHA-mediated T-reg-induction could act as a potential mechanism of action (MOA) for controlling inflammatory CF-lung disease.

**Fig. 6:**
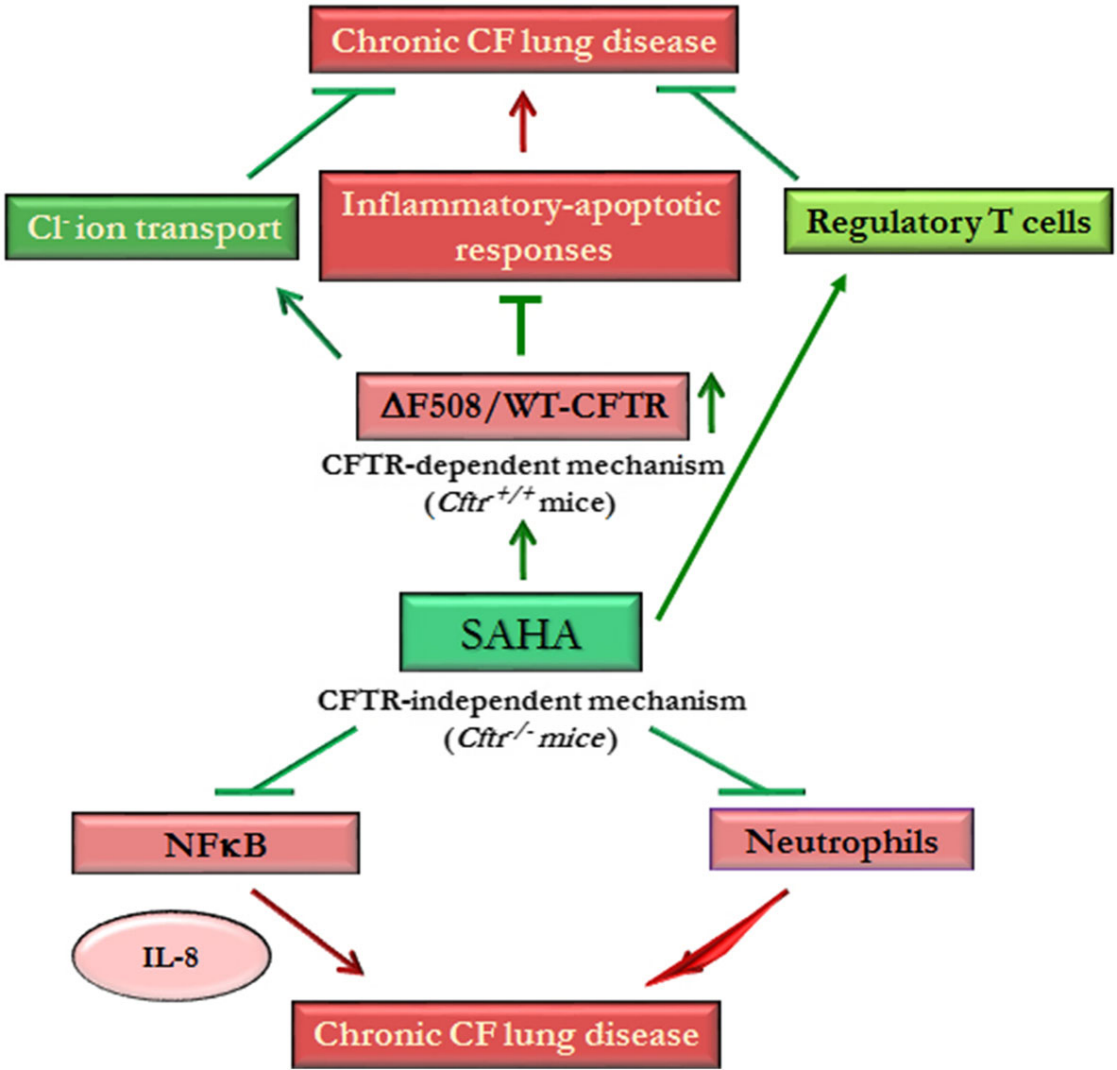
SAHA induces a protective anti-inflammatory triad for controlling chronic CF-lung disease. Schematic showing the potential protective mechanisms by which SAHA controls airway inflammation by both CFTR-dependent (*Cftr*^*+/+*^) and independent (*Cftr*^*−/−*^) mechanisms. Treatment with SAHA induces, a ‘protective triad’ by suppressing NFκB-mediated inflammatory signaling, neutrophil recruitment and/or activation, and augmentation of the immunosuppressive T-reg cells. This is specifically important, as CFTR expression/function can be inhibited by the presence of *Pa-*infection or neutrophil elastase. In this scenario, SAHA treatment would assist in controlling inflammatory-apoptotic responses via its ability to rescue CFTR to the plasma membrane. Moreover, similar to its mechanism of action in the presence of CFTR, SAHA treatment also controls NFκB-mediated inflammatory signaling and neutrophil chemotaxis/activation in the absence of CFTR. Thus, selective HDACi strategy could be utilized to design a novel therapeutic for effectively reverting the chronic-CF lung disease, as it augments innate and adaptive immune responses, and ΔF508-CFTR function. The rescue of disease from chronic stages requires correction of the underlying CFTR-protein defect as well as immune response augmentation, which can be achieved by the proposed therapeutic intervention strategy

Although, the use of anti-inflammatory treatment strategies seems promising, their efficacy is restricted to symptomatic control of CF airway inflammation rather than targeting the basic genetic defect that leads to the loss of the functional membrane localized CFTR-protein. Previous studies have indicated that SAHA also acts as a proteostasis-modulator that may help restore the membrane expression and function of misfolded ΔF508-CFTR [3, 17, 34], warranting its further preclinical evaluation for treating chronic CF-lung disease. In support of this concept, we demonstrate that HDACi by SAHA and Trichostatin-A, indeed delays the degradation of ΔF508-CFTR protein, compared to the untreated control (Fig. 5c). Although, a single recent study suggests that SAHA may not significantly rescue membrane CFTR levels but data from this study is inconclusive due to insuffiecient number of replicates and experimental variation, where SAHA mediated CFTR increase was observed in some cellular models but not other [43]. Moreover, the same study also suggests that SAHA treatment decreases CFTR mRNA levels and thus cannot be used as an efficient CFTR corrector. In contrast, in our observation and studies from other groups [3, 17], neither SAHA nor any other HDACi decreases CFTR mRNA levels. Additionally, we also evaluated the pre-clinical therapeutic efficacy of SAHA in controlling *Pa*-LPS induced neutrophil activity and lung inflammation in *Cftr*^*+/+*^ and *Cftr*^−/−^-gut-corrected (Figs. 4, 5a and b, Fig. 6) murine models of airway-inflammation and chronic-CF lung disease [16]. Our finding that SAHA controls *Pa*-LPS induced lung inflammation and neutrophil activity in *Cftr*^*+/+*^ mice might have interesting implications in ΔF508-CF and COPD subjects with acquired CFTR-dysfunction, where SAHA can augment CFTR function as well as control inflammatory response by CFTR dependent and independent mechanisms (Fig. 6). In support of these findings, previous studies have clearly demonstrated the key role of membrane-localized WT-CFTR in controlling chronic inflammatory signaling in CF and other chronic inflammatory lung diseases [1, 2, 8, 16, 22, 44]. Moreover, it is also important to note that both *Pa-*infection and neutrophil elastase can negatively affect CFTR expression and/or function [45–47]. Thus, it is plausible that rescue of functional CFTR-activity is one of the mechanisms by which SAHA protects against *Pa*-LPS induced lung inflammation in *Cftr*^*+/+*^ mice. This suggests an additional potential therapeutic application of SAHA in controlling intermittent and stable *Pa-*exacerbations in both ΔF508-CF and COPD subjects [46], that warrants further clinical evaluation. In addition, we used *Pa*-LPS induced chronic lung inflammatory (*Cftr*^*−/−*^) murine model to verify CFTR-independent anti-inflammatory potential of SAHA, which demonstrates its therapeutic effectiveness in controlling CF-inflammatory lung disease, irrespective of mutant-CFTR rescue or correction. To the best of our knowledge, this is the first report demonstrating the efficacy and the mechanism of action of HDACi, such as SAHA in controlling CF-related lung inflammation. Finally, identification of specific HDACs that control CFTR processing [3, 17] and lung disease, would lead to the development of selective HDAC-inhibitor drugs. As a proof of concept, HDAC6 (Tubacin) [48] and HDAC7 [3, 17, 48] inhibitors have known potential application in rescuing misfolded-CFTR protein from proteasomal degradation or aggresome-accumulation.

## Conclusions

In conclusion, proposed SAHA-mediated CF-lung disease intervention can benefit from multiple mechanism of actions such as (1) an anti-inflammatory, (2) T-reg inducer and (3) ΔF508-CFTR corrector drug (Fig. 6). Thus, further clinical evaluation of this potent pharmaceutical strategy is warranted, and we anticipate that using the specific HDAC-inhibitors [3, 17] and novel nano-based drug delivery system [49], that we recently described, can further enhance its therapeutic potency in reverting the CF-lung disease.

## Additional files

Additional file 1: Figure S1. Selective HDAC inhibition controls ER-stress activity. **(A)** HEK293 cells were transiently transfected with two different HDAC6 shRNA constructs or control plasmid and the cell lysates were immunoblotted for HDAC6 and β-actin. The immunoblot verifies the >2 fold knockdown efficiency of HDACshRNA for experiment shown in B. **(B)** HEK293 cells were transiently transfected with a secretory gaussia reporter plasmid and/or HDAC6 shRNA. After 6 h of transfection cells were treated with CSE (cigarette smoke extract, as ER stress activator), SAHA (10 μM), or Tubacin (10 μM) after 6 h. At 72 (B, upper panel) or 24 (B, lower panel) hours, supernatants were collected and read with the aforementioned Dual Luciferase Reporter System to determine ER-stress activity. The data (mean ± SD of triplicate samples) shows that CSE induced ER-stress activity (*p* < 0.05) is significantly controlled by Class II HDAC inhibitor, (SAHA), selective HDAC6 inhibitor (Tubacin, B-upper panel) and HDAC6 shRNA (B-lower panel) suggesting the therapeutic potential of selective HDACi in controlling CF-related ER-stress response. **(C)** The *Cftr*^*+/+*^ mice were exposed to sub-chronic cigarette smoke (sc-CS, 8 weeks) and i.t. instilled with SAHA (50 μg/mouse, three total doses with one day interval before the termination of the experiment). The BALF cells were harvested and analyzed for CD4+ (CD4-PE antibody) and FoxP3+ [(FoxP3 (Rabbit polyclonal primary Ab)-Anti-Rabbit-FITC (secondary Ab)] cells by flow cytometry using the BD FACS Caliber instrument. The data indicates that SAHA treatment induces the levels of FoxP3+ T regs to counteract the sc-CS mediated airway inflammation. (JPEG 259 kb)

## Abbreviations

NFκB: Nuclear factor kappa-light-chain-enhancer of activated B cells
BALF: Bronchoalveolar lavage fluid
CF: Cystic fibrosis
CFTR: Cystic fibrosis transmembrane conductance regulator
COPD: Chronic obstructive pulmonary disease
CS: Cigarette smoke
CSE: Cigarette smoke extract
ER stress: Endoplasmic reticulum stress
HDAC: Histone deacetylase
HEK: Human embryonic kidney cells
i.t.: Intra-tracheal
IBD: Inflammatory bowel disease
IL-6/8: Interleukin-6/8
MPO: Myeloperoxidase
Nrf2: Nuclear factor (erythroid-derived 2)-like 2
*Pa*-LPS: *Pseudomonas aeruginosa* lipopolysaccharide
ROS: Reactive oxygen species
SAHA: Suberoylanilide hydroxamic acid
sc-CS: Sub-chronic cigarette smoke
SDS-PAGE: Sodium dodecyl sulfate polyacrylamide gel electrophoresis
Th: T-helper cells
T-regs: Regulatory T cells
TSA: Trichostatin-A
UPR: Unfolded protein response
UPS: Ubiquitin proteasome system
