# Supplementary figures and images for: Inhibition of histone-deacetylase activity rescues inflammatory cystic fibrosis lung disease by modulating innate and adaptive immune responses
Source: Respir Res. 2018 Jan 4;19:2. doi: 10.1186/s12931-017-0705-8 (PMC5755330; doi:10.1186/s12931-017-0705-8)

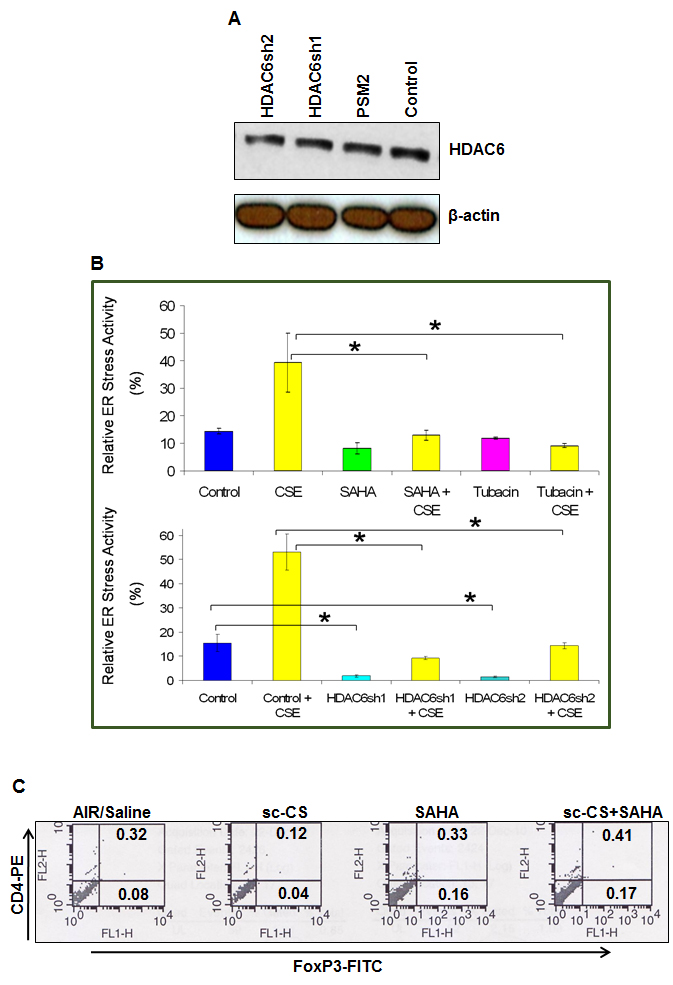

Supplement: Supplementary file 1 — Selective HDAC inhibition controls ER-stress activity. (A) HEK293 cells were transiently transfected with two different HDAC6 shRNA constructs or control plasmid and the cell lysates were immunoblotted for HDAC6 and β-actin. The immunoblot verifies the >2 fold knockdown efficiency of HDACshRNA for experiment shown in B. (B) HEK293 cells were transiently transfected with a secretory gaussia reporter plasmid and/or HDAC6 shRNA. After 6 h of transfection cells were treated with CSE (cigarette smoke extract, as ER stress activator), SAHA (10 μM), or Tubacin (10 μM) after 6 h. At 72 (B, upper panel) or 24 (B, lower panel) hours, supernatants were collected and read with the aforementioned Dual Luciferase Reporter System to determine ER-stress activity. The data (mean ± SD of triplicate samples) shows that CSE induced ER-stress activity (p < 0.05) is significantly controlled by Class II HDAC inhibitor, (SAHA), selective HDAC6 inhibitor (Tubacin, B-upper panel) and HDAC6 shRNA (B-lower panel) suggesting the therapeutic potential of selective HDACi in controlling CF-related ER-stress response. (C) The Cftr+/+ mice were exposed to sub-chronic cigarette smoke (sc-CS, 8 weeks) and i.t. instilled with SAHA (50 μg/mouse, three total doses with one day interval before the termination of the experiment). The BALF cells were harvested and analyzed for CD4+ (CD4-PE antibody) and FoxP3+ [(FoxP3 (Rabbit polyclonal primary Ab)-Anti-Rabbit-FITC (secondary Ab)] cells by flow cytometry using the BD FACS Caliber instrument. The data indicates that SAHA treatment induces the levels of FoxP3+ T regs to counteract the sc-CS mediated airway inflammation. (JPEG 259 kb) [file 12931_2017_705_MOESM1_ESM.jpg]
